# Supplementary material for: Comparative Genomics Applied to Systematically Assess Pathogenicity Potential in Shiga Toxin-Producing Escherichia coli O145:H28
Source: Microorganisms. 2022 Apr 21;10(5):866. doi: 10.3390/microorganisms10050866 (PMC9144400; doi:10.3390/microorganisms10050866)
Supplement: Supplementary file 1 [file microorganisms-10-00866-s001.zip › Figure S1.pdf]

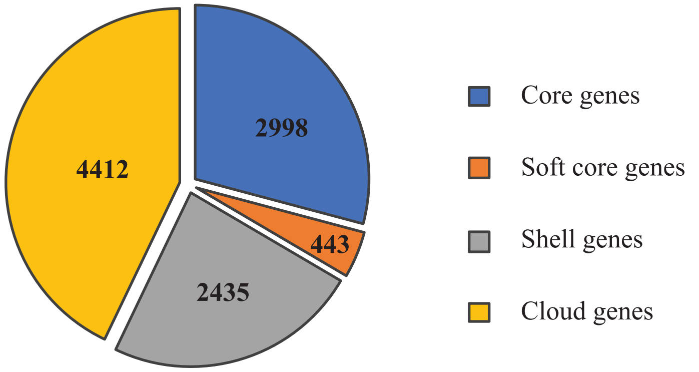

Supplemental Figure S1. Genomic subset genes in STEC. 22 STEC genomes, including 19 O145:H28 and three STEC reference genomes as detailed in Table 1, were used for comparative genomic analyses. The core and accessory genes were calculated in Roary as detailed in Materials and Methods section. The core genes refer to all genes shared by all input genomes; the soft core genes refer to all genes present in any 21 input genomes; the shell genes refer to all genes present in at least four but less than 21 input genomes; the cloud genes refer to all genes present in less than four input genomes.
