# Supplementary material for: Comparative Genomics Applied to Systematically Assess Pathogenicity Potential in Shiga Toxin-Producing Escherichia coli O145:H28
Source: Microorganisms. 2022 Apr 21;10(5):866. doi: 10.3390/microorganisms10050866 (PMC9144400; doi:10.3390/microorganisms10050866)
Supplement: Supplementary file 1 [file microorganisms-10-00866-s001.zip › Figure S2.pdf]

Figure S2. Comparative analyses of PAIs in STEC strains. A: Relatedness of LEE islands; B: Relatedness of the OI-122 islands; C: Relatedness of the OI-57 islands; D: Relatedness of the TRIs. The DNA sequences of 22 LEE islands (A), 21 OI-122 islands (B), 20 OI-57 islands (C), and 24 TRIs (D) were aligned in Geneious Prime®, correspondingly, using Clustal Omega, followed by constructing a consensus tree using Jukes-Cantor Genetic Distance Model and Neighbor-Joining Tree Build Method without an outgroup as detailed in the Materials and Methods section.

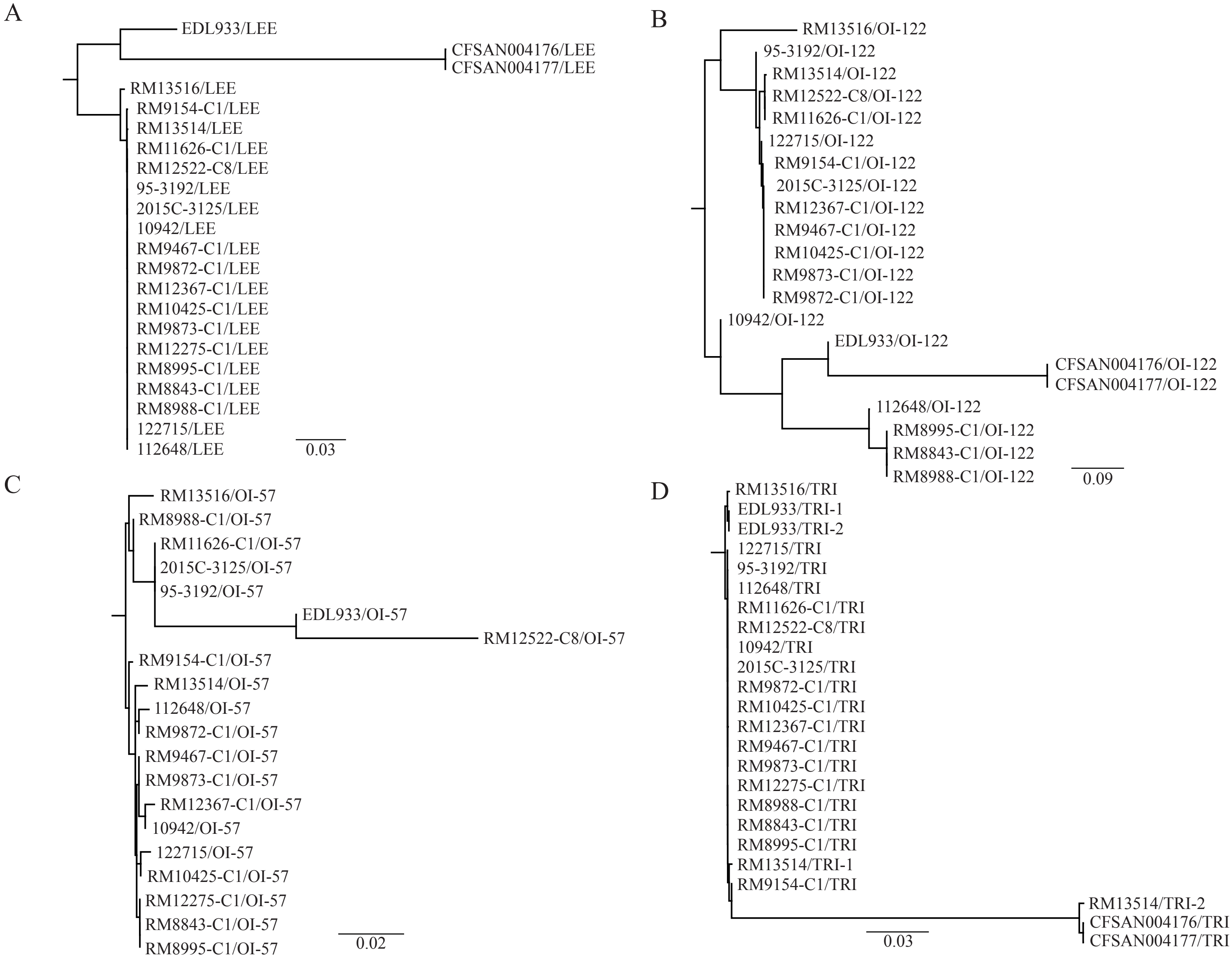
A
EDL933/LEE
CFSAN004176/LEE
CFSAN004177/LEE
RM13516/LEE
RM9154-C1/LEE
RM13514/LEE
RM11626-C1/LEE
RM12522-C8/LEE
95-3192/LEE
2015C-3125/LEE
10942/LEE
RM9467-C1/LEE
RM9872-C1/LEE
RM12367-C1/LEE
RM10425-C1/LEE
RM9873-C1/LEE
RM12275-C1/LEE
RM8995-C1/LEE
RM8843-C1/LEE
RM8988-C1/LEE
122715/LEE
112648/LEE
0.03
B
RM13516/OI-122
95-3192/OI-122
RM13514/OI-122
RM12522-C8/OI-122
RM11626-C1/OI-122
122715/OI-122
RM9154-C1/OI-122
2015C-3125/OI-122
RM12367-C1/OI-122
RM9467-C1/OI-122
RM10425-C1/OI-122
RM9873-C1/OI-122
RM9872-C1/OI-122
10942/OI-122
EDL933/OI-122
CFSAN004176/OI-122
CFSAN004177/OI-122
112648/OI-122
RM8995-C1/OI-122
RM8843-C1/OI-122
RM8988-C1/OI-122
0.09
C
RM13516/OI-57
RM8988-C1/OI-57
RM11626-C1/OI-57
2015C-3125/OI-57
95-3192/OI-57
EDL933/OI-57
RM12522-C8/OI-57
RM9154-C1/OI-57
RM13514/OI-57
112648/OI-57
RM9872-C1/OI-57
RM9467-C1/OI-57
RM9873-C1/OI-57
RM12367-C1/OI-57
10942/OI-57
122715/OI-57
RM10425-C1/OI-57
RM12275-C1/OI-57
RM8843-C1/OI-57
RM8995-C1/OI-57
0.02
D
RM13516/TRI
EDL933/TRI-1
EDL933/TRI-2
122715/TRI
95-3192/TRI
112648/TRI
RM11626-C1/TRI
RM12522-C8/TRI
10942/TRI
2015C-3125/TRI
RM9872-C1/TRI
RM10425-C1/TRI
RM12367-C1/TRI
RM9467-C1/TRI
RM9873-C1/TRI
RM12275-C1/TRI
RM8988-C1/TRI
RM8843-C1/TRI
RM8995-C1/TRI
RM13514/TRI-1
RM9154-C1/TRI
RM13514/TRI-2
CFSAN004176/TRI
CFSAN004177/TRI
0.03
