# Supplementary material for: Comparative Genomics Applied to Systematically Assess Pathogenicity Potential in Shiga Toxin-Producing Escherichia coli O145:H28
Source: Microorganisms. 2022 Apr 21;10(5):866. doi: 10.3390/microorganisms10050866 (PMC9144400; doi:10.3390/microorganisms10050866)
Supplement: Supplementary file 1 [file microorganisms-10-00866-s001.zip › Table S1.pdf]

Table S1. *E. coli* virulence genes[1].

VFclass	Virulence factors	Genes2	Strain/Pathotype	GenBank Accession/Replicon	Locus ID	Genomic subset[3]	EDL933	CFSAN004176	CFSAN004177	RM13514	RM13516	10942	112648	122715	95-3192[4]	2015C-0315	RM8843-C1	RM8988-C1	RM8995-C1	RM9154-C1	RM9467-C1	RM9872-C1	RM9873-C1	RM10425-C1	RM11626-C1	RM12275-C1	RM12367-C1	RM12522-C8
Adherence	AAF/II fimbriae	*aafA*	*E. coli* O44:H18 str. 042/EAEC	NC_017627/Plasmid pAA	EC042_pAA048	NA	0	0	0	0	0	0	0	0	0	0	0	0	0	0	0	0	0	0	0	0	0	0
		aafB			EC042_pAA030	NA	0	0	0	0	0	0	0	0	0	0	0	0	0	0	0	0	0	0	0	0	0	0
		aafC			EC042_pAA031	NA	0	0	0	0	0	0	0	0	0	0	0	0	0	0	0	0	0	0	0	0	0	0
		aafD			EC042_pAA046	NA	0	0	0	0	0	0	0	0	0	0	0	0	0	0	0	0	0	0	0	0	0	0
	AAF/III fimbriae	*aggA*	*E. coli* O104:H4 str. 2011C-3493 /StxEAEC	NC_018666 /Plasmid pAA-EA11	O3K_26197	NA	0	0	0	0	0	0	0	0	0	0	0	0	0	0	0	0	0	0	0	0	0	0
		aggB			O3K_26202	NA	0	0	0	0	0	0	0	0	0	0	0	0	0	0	0	0	0	0	0	0	0	0
		aggC			O3K_26207	NA	0	0	0	0	0	0	0	0	0	0	0	0	0	0	0	0	0	0	0	0	0	0
		aggD			O3K_26212	NA	0	0	0	0	0	0	0	0	0	0	0	0	0	0	0	0	0	0	0	0	0	0
		aggR			O3K_26097	NA	0	0	0	0	0	0	0	0	0	0	0	0	0	0	0	0	0	0	0	0	0	0
	Afimbrial adhesin AFA-I	*afaA*	*E. coli* str. VR50 /UPEC	NZ_CP011134/Chromosome	ECVR50_3238	NA	0	0	0	0	0	0	0	0	0	0	0	0	0	0	0	0	0	0	0	0	0	0
		afaB			ECVR50_3237	NA	0	0	0	0	0	0	0	0	0	0	0	0	0	0	0	0	0	0	0	0	0	0
		afaC			ECVR50_3236	NA	0	0	0	0	0	0	0	0	0	0	0	0	0	0	0	0	0	0	0	0	0	0
		afaD			ECVR50_3235	NA	0	0	0	0	0	0	0	0	0	0	0	0	0	0	0	0	0	0	0	0	0	0
		afaE			ECVR50_3233	NA	0	0	0	0	0	0	0	0	0	0	0	0	0	0	0	0	0	0	0	0	0	0
		draP			ECVR50_3234	NA	0	0	0	0	0	0	0	0	0	0	0	0	0	0	0	0	0	0	0	0	0	0
	CFA/I fimbriae	*cfaA*	*E. coli* O103:H2 str. 12009/EHEC	NC_013353/Chromosome	ECO103_3842	A	0	1	1	0	0	0	0	0	0	0	0	0	0	0	0	0	0	0	0	0	0	0
		cfaB			ECO103_3841	A	0	1	1	0	0	0	0	0	0	0	0	0	0	0	0	0	0	0	0	0	0	0
		cfaC			ECO103_3840	A	0	1	1	0	0	0	0	0	0	0	0	0	0	0	0	0	0	0	0	0	0	0
		cfaD/cfaE			ECO103_3839	A	0	1	1	0	0	0	0	0	0	0	0	0	0	0	0	0	0	0	0	0	0	0
	Curli fimbriae	*csgD*	*E. coli* O157:H7 str. EDL933/EHEC	NC_002655/Chromosome	Z1673	C	1	1	1	1	1	1	1	1	1	1	1	1	1	1	1	1	1	1	1	1	1	1
		csgE			Z1672	C	1	1	1	1	1	1	1	1	1	1	1	1	1	1	1	1	1	1	1	1	1	1
		csgF			Z1671	C	1	1	1	1	1	1	1	1	1	1	1	1	1	1	1	1	1	1	1	1	1	1
		csgG			Z1670	C	1	1	1	1	1	1	1	1	1	1	1	1	1	1	1	1	1	1	1	1	1	1
		csgA			Z1676	C	1	1	1	1	1	1	1	1	1	1	1	1	1	1	1	1	1	1	1	1	1	1
		csgB			Z1675	C	1	1	1	1	1	1	0	1	1	1	1	1	1	1	1	1	1	1	1	1	1	1
		csgC			Z1677	C	1	1	1	1	1	1	1	1	1	1	1	1	1	1	1	1	1	1	1	1	1	1
	Dispersin	*aap*	*E. coli* O44:H18 str. 042/EAEC	NC_017627/Plasmid pAA	EC042_pAA055	NA	0	0	0	0	0	0	0	0	0	0	0	0	0	0	0	0	0	0	0	0	0	0
	E. coli common pilus (ECP)	*ecpA*	*E. coli* O104:H4 str. 2011C-3493 /StxEAEC	NC_018658/Chromosome	O3K_20025	C	1	1	1	1	1	1	1	1	1	1	1	1	1	1	1	1	1	1	1	1	1	1
		ecpB			O3K_20030	C	1	1	1	1	1	1	1	1	1	1	1	1	1	1	1	1	1	1	1	1	1	1
		ecpC			O3K_20035	C	1	1	1	1	1	1	1	1	1	1	1	1	1	1	1	1	1	1	1	1	1	1
		ecpD			O3K_20040	C	1	1	1	1	1	1	1	1	1	1	1	1	1	1	1	1	1	1	1	1	1	1
		ecpE			O3K_20045	C	1	1	0	1	1	1	1	1	1	1	1	1	1	1	0	1	1	1	1	1	1	1
		ecpR			O3K_20020	C	1	1	1	1	1	1	1	1	1	1	1	1	1	1	1	1	1	1	1	1	1	1
	E.coli laminin-binding fimbriae (ELF)	*elfA*	*E.coli* O26:H11 str. 11368/EHEC	NC_013361/Chromosome	ECO26_1065	C	1	1	1	1	1	1	1	1	1	1	1	1	1	1	1	1	1	1	1	1	1	1
		elfC			ECO26_1067	C	0	1	0	1	1	1	1	1	1	0	0	1	0	1	1	1	1	1	1	0	0	1
		elfD			ECO26_1066	C	1	1	0	1	1	1	1	1	1	1	1	1	1	1	1	1	1	1	1	1	1	1
		elfG			ECO26_1068	C	0	1	0	1	1	1	1	1	0	1	1	1	1	1	1	1	1	1	1	1	0	1
	Adhesin FdeC	*eaeH*	*E. coli* O157:H7 str. EDL933/EHEC	NC_002655/Chromosome	Z0375	A	1	1	1	1	0	1	1	1	1	1	1	1	0	1	1	1	0	1	1	0	1	1
	EtpA	*etpA*	*E. coli* O78:H11:K80 str. H10407/ETEC	NC_017724/Plasmid p948	ETEC_p948_0110	NA	0	0	0	0	0	0	0	0	0	0	0	0	0	0	0	0	0	0	0	0	0	0
	F1C fimbriae	*focA*	*E. coli* str. CFT073/UPEC	NC_004431/Chromosome	c1239	NA	0	0	0	0	0	0	0	0	0	0	0	0	0	0	0	0	0	0	0	0	0	0
		focC			c1241	NA	0	0	0	0	0	0	0	0	0	0	0	0	0	0	0	0	0	0	0	0	0	0
		focD			c1242	NA	0	0	0	0	0	0	0	0	0	0	0	0	0	0	0	0	0	0	0	0	0	0
		focF			c1243	NA	0	0	0	0	0	0	0	0	0	0	0	0	0	0	0	0	0	0	0	0	0	0
		focG			c1244	NA	0	0	0	0	0	0	0	0	0	0	0	0	0	0	0	0	0	0	0	0	0	0
		focH			c1245	NA	0	0	0	0	0	0	0	0	0	0	0	0	0	0	0	0	0	0	0	0	0	0
		focI			c1240	NA	0	0	0	0	0	0	0	0	0	0	0	0	0	0	0	0	0	0	0	0	0	0
	Hemorrhagic *E.coli* pilus (HCP)	*hcpA*	*E. coli* O157:H7 str. EDL933/EHEC	NC_002655/Chromosome	Z0118	C	1	1	1	1	1	1	1	1	1	1	1	1	1	1	1	1	1	1	1	1	1	1
		hcpB			Z0117	C	1	1	1	1	1	1	1	1	1	1	1	1	1	1	1	1	1	1	1	1	1	1
		hcpC			Z0116	C	1	1	1	1	1	1	1	1	1	1	1	1	1	1	1	1	1	1	1	1	1	1
	Intimin	*eae*	*E. coli* O157:H7 str. EDL933/EHEC	NC_002655/Chromosome	Z5110	C	1	1	1	1	1	1	1	1	0	1	1	1	1	1	1	1	1	1	1	0	1	1
	K88 fimbriae	*faeC*	*E. coli* str. UMNK88/ETEC	NC_017639/Plasmid pUMNK88_K88	UMNK88_pK887	NA	0	0	0	0	0	0	0	0	0	0	0	0	0	0	0	0	0	0	0	0	0	0
		faeD			UMNK88_pK888	NA	0	0	0	0	0	0	0	0	0	0	0	0	0	0	0	0	0	0	0	0	0	0
		faeE			UMNK88_pK889	NA	0	0	0	0	0	0	0	0	0	0	0	0	0	0	0	0	0	0	0	0	0	0
		faeF			UMNK88_pK8810	NA	0	0	0	0	0	0	0	0	0	0	0	0	0	0	0	0	0	0	0	0	0	0
		faeG			UMNK88_pK8811	NA	0	0	0	0	0	0	0	0	0	0	0	0	0	0	0	0	0	0	0	0	0	0
		faeH			UMNK88_pK8812	NA	0	0	0	0	0	0	0	0	0	0	0	0	0	0	0	0	0	0	0	0	0	0
		faeI			UMNK88_pK8813	NA	0	0	0	0	0	0	0	0	0	0	0	0	0	0	0	0	0	0	0	0	0	0
		faeJ			UMNK88_pK8814	NA	0	0	0	0	0	0	0	0	0	0	0	0	0	0	0	0	0	0	0	0	0	0
	P fimbriae	*papA*	*E. coli* str. UM146/AIEC	NC_017632/Chromosome	UM146_21685	NA	0	0	0	0	0	0	0	0	0	0	0	0	0	0	0	0	0	0	0	0	0	0
		papB			UM146_21690	NA	0	0	0	0	0	0	0	0	0	0	0	0	0	0	0	0	0	0	0	0	0	0
		papC			UM146_21675	A	0	1	1	0	0	0	0	0	0	0	0	0	0	0	0	0	0	0	0	0	0	0
		papD			UM146_21670	A	0	1	1	0	0	0	0	0	0	0	0	0	0	0	0	0	0	0	0	0	0	0
		papE			UM146_21655	NA	0	0	0	0	0	0	0	0	0	0	0	0	0	0	0	0	0	0	0	0	0	0
		papF			UM146_21650	NA	0	0	0	0	0	0	0	0	0	0	0	0	0	0	0	0	0	0	0	0	0	0
		papG			UM146_21645	NA	0	0	0	0	0	0	0	0	0	0	0	0	0	0	0	0	0	0	0	0	0	0
		papH			UM146_21680	NA	0	0	0	0	0	0	0	0	0	0	0	0	0	0	0	0	0	0	0	0	0	0
		papI			UM146_21695	NA	0	0	0	0	0	0	0	0	0	0	0	0	0	0	0	0	0	0	0	0	0	0
		papJ			UM146_21665	NA	0	0	0	0	0	0	0	0	0	0	0	0	0	0	0	0	0	0	0	0	0	0
		papK			UM146_21660	NA	0	0	0	0	0	0	0	0	0	0	0	0	0	0	0	0	0	0	0	0	0	0
		papX			UM146_21640	NA	0	0	0	0	0	0	0	0	0	0	0	0	0	0	0	0	0	0	0	0	0	0
	Porcine attaching-effacing associated protein	*paa*	*E. coli* O103:H2 str. 12009/EHEC	NC_013353/Chromosome	ECO103_1701	C	1	1	1	2	1	1	1	1	1	1	1	1	1	1	1	1	1	1	1	1	1	1
	S fimbriae	*sfaA*	*E. coli* str. UM146/AIEC	NC_017632/Chromosome	UM146_12360	NA	0	0	0	0	0	0	0	0	0	0	0	0	0	0	0	0	0	0	0	0	0	0
		sfaB			UM146_12365	NA	0	0	0	0	0	0	0	0	0	0	0	0	0	0	0	0	0	0	0	0	0	0
		sfaC			UM146_12370	NA	0	0	0	0	0	0	0	0	0	0	0	0	0	0	0	0	0	0	0	0	0	0
		sfaD			UM146_12355	NA	0	0	0	0	0	0	0	0	0	0	0	0	0	0	0	0	0	0	0	0	0	0

		sfaE			UM146_12350	NA	0	0	0	0	0	0	0	0	0	0	0	0	0	0	0	0	0	0	0	0	0	0
		sfaF			UM146_12345	NA	0	0	0	0	0	0	0	0	0	0	0	0	0	0	0	0	0	0	0	0	0	0
		sfaG			UM146_12340	NA	0	0	0	0	0	0	0	0	0	0	0	0	0	0	0	0	0	0	0	0	0	0
		sfaH			UM146_12330	NA	0	0	0	0	0	0	0	0	0	0	0	0	0	0	0	0	0	0	0	0	0	0
		sfaS			UM146_12335	NA	0	0	0	0	0	0	0	0	0	0	0	0	0	0	0	0	0	0	0	0	0	0
		sfaY			UM146_12325	NA	0	0	0	0	0	0	0	0	0	0	0	0	0	0	0	0	0	0	0	0	0	0
	ToxB	*toxB*	*E. coli* O157:H7 str. EDL933/EHEC	NC_007414/Plasmid pO157	L7095	A	1	0	0	1	0	1	1	1	0	0	1	1	0	1	0	0	0	1	0	0	0	0
	Type I fimbriae	*fimA*	*E. coli* O157:H7 str. EDL933/EHEC	NC_002655/Chromosome	Z5912	C	1	1	1	1	1	1	1	1	1	1	1	1	1	1	1	1	1	1	1	1	1	1
		fimB			Z5910	C	1	1	1	1	0	1	1	1	1	1	1	1	1	1	1	1	1	1	1	1	1	1
		fimC			Z5914	C	1	1	1	1	1	1	1	1	1	1	1	1	1	1	1	1	1	1	1	1	1	1
		fimD			Z5915	C	1	1	1	1	1	1	1	1	1	0	1	1	1	1	1	1	1	1	1	1	1	1
		fimE			Z5911	C	1	1	1	1	1	1	1	1	1	1	1	1	1	1	1	1	1	1	1	1	1	1
		fimF			Z5916	C	1	1	1	1	1	1	1	1	1	1	1	1	1	1	1	1	1	1	1	1	1	1
		fimG			Z5917	C	1	1	1	1	1	1	1	1	1	1	1	1	1	1	1	1	1	1	1	1	1	1
		fimH			Z5918	C	1	1	1	1	1	1	1	1	1	1	1	1	1	1	1	1	1	1	1	1	1	1
		fimI			Z5913	C	1	1	1	1	1	1	1	1	1	1	1	1	1	1	1	1	1	1	1	1	1	1
Auto-transporter	AIDA-I type	*tibA*	*E. coli* O78:H11:K80 str. H10407/ETEC	NC_017633/Chromosome	ETEC_2141	NA	0	0	0	0	0	0	0	0	0	0	0	0	0	0	0	0	0	0	0	0	0	0
	AIDA-I	*aidA*	*E. coli* O25b:H4-ST131/UPEC	NZ_HG941718/Chromosome	EC958_4121	NA	0	0	0	0	0	0	0	0	0	0	0	0	0	0	0	0	0	0	0	0	0	0
	AatA	*aatA*	*E. coli* APEC str. O1/APEC	NC_009837/Plasmid pAPEC-O1-ColBM	APECO1_ O1CoBM96	NA	0	0	0	0	0	0	0	0	0	0	0	0	0	0	0	0	0	0	0	0	0	0
	Antigen 43	*agn43*	*E. coli* O104:H4 str. 2011C-3493/StxEAEC	NC_018658/Chromosome	O3K_04220	A	0	0	0	1	2	0	1	1	1	0	1	1	1	0	1	1	1	1	1	0	1	1
	Cah	*cah*	*E. coli* O157:H7 str. EDL933/EHEC	NC_002655/Chromosome	Z1211	A	2	0	0	0	0	1	1	1	1	1	1	1	1	1	0	1	0	1	1	0	1	1
	Contact-dependent inhibition CDI system	*cdiA*	*E. coli* str. UM146/AIEC	NC_017632/Chromosome	UM146_21955	NA	0	0	0	0	0	0	0	0	0	0	0	0	0	0	0	0	0	0	0	0	0	0
		cdiB			UM146_21960	NA	0	0	0	0	0	0	0	0	0	0	0	0	0	0	0	0	0	0	0	0	0	0
	EhaA	*ehaA*	*E. coli* O157:H7 str. EDL933/EHEC	NC_002655/Chromosome	Z0402	C	1	1	1	1	1	1	1	1	1	1	1	1	1	0	1	1	1	1	1	0	1	1
	EhaB	*ehaB*			Z0469	C	1	1	1	1	1	0	1	1	1	1	1	1	1	1	1	1	1	1	1	1	1	1
	Enteroaggregative immunoglobulin repeat protein	*air/eaeX*	*E. coli* O44:H18 042/EAEC	NC_017626/Chromosome	EC042_4012	NA	0	0	0	0	0	0	0	0	0	0	0	0	0	0	0	0	0	0	0	0	0	0
	EspC	*espC*	*E. coli* O127:H6 str. E2348/69/EPEC	NC_011601/Chromosome	E2348C_2915	NA	0	0	0	0	0	0	0	0	0	0	0	0	0	0	0	0	0	0	0	0	0	0
	EspI	*espI*	*E. coli* O103:H2 str. 12009/EHEC	NC_013353/Chromosome	ECO103_3805	A	0	1	2	1	0	0	0	0	0	0	0	0	0	0	0	0	0	0	0	0	0	0
	EspP	*espP*	*E. coli* O157:H7 str. EDL933/EHEC	NC_007414/Plasmid pO157	L7020	A	1	0	0	1	0	1	1	1	0	0	1	1	1	1	1	1	1	1	1	0	1	1
	Pet	*pet*	*E. coli* O104:H4 str. 2011C-3493 /StxEAEC	NC_018658/Chromosome	O3K_23005	NA	0	0	0	0	0	0	0	0	0	0	0	0	0	0	0	0	0	0	0	0	0	0
	Pic	*pic*			O3K_23140	NA	0	0	0	0	0	0	0	0	0	0	0	0	0	0	0	0	0	0	0	0	0	0
	Sat	*sat*	*E. coli* O25b:H4-ST131/UPEC	NZ_HG941718/Chromosome	EC958_3278	NA	0	0	0	0	0	0	0	0	0	0	0	0	0	0	0	0	0	0	0	0	0	0
	Temperature-sensitive hemagglutinin	*tsh*			EC958_4611	NA	0	0	0	0	0	0	0	0	0	0	0	0	0	0	0	0	0	0	0	0	0	0
	UpaG adhesin	*upaG/ehaG*			EC958_4009	C	1	1	1	1	1	0	1	1	1	1	1	1	1	1	1	1	1	1	1	1	1	1
	UpaH	*upaH*			EC958_1689	NA	0	0	0	0	0	0	0	0	0	0	0	0	0	0	0	0	0	0	0	0	0	0
	Vacuolating autotransporter gene	*vat*	*E. coli* str. UM146/AIEC	NC_017632/Chromosome	UM146_15930	NA	0	0	0	0	0	0	0	0	0	0	0	0	0	0	0	0	0	0	0	0	0	0
Invasion	Invasion of brain endothelial cells (Ibes)	*ibeA*	*E. coli* str. UM146/AIEC	NC_017632/Chromosome	UM146_22380	NA	0	0	0	0	0	0	0	0	0	0	0	0	0	0	0	0	0	0	0	0	0	0
		ibeB			UM146_14655	C	1	1	1	1	1	1	1	1	1	1	1	1	1	1	1	1	1	1	1	1	1	1
		ibeC			UM146_20030	C	2	1	1	2	2	2	2	2	1	1	2	2	2	2	2	2	2	2	2	2	2	2
	Tia/Hek	*tia*			UM146_21625	NA	0	0	0	0	0	0	0	0	0	0	0	0	0	0	0	0	0	0	0	0	0	0
Iron uptake	Aerobactin siderophore	*iucA*	*E. coli* O104:H4 str. 2011C-3493 /StxEAEC	NC_018658/Chromosome	O3K_23085	A	0	0	0	1	1	1	1	1	1	1	1	1	1	0	1	1	1	1	1	1	1	1
		iucB			O3K_23080	A	0	0	0	1	1	1	1	1	1	1	1	1	1	0	1	1	1	1	1	1	1	1
		iucC			O3K_23075	A	0	0	0	1	1	1	1	1	1	1	1	1	1	0	0	0	0	0	1	1	0	1
		iucD			O3K_23070	A	0	0	0	1	1	1	1	1	1	1	1	1	1	0	1	1	1	1	0	1	1	0
		iutA			O3K_23065	A	0	0	0	1	1	1	1	1	1	1	1	1	1	0	1	1	1	1	1	1	1	1
	Heme uptake	*chuA*	*E. coli* O157:H7 str. EDL933/EHEC	NC_002655/Chromosome	Z4911	A	1	0	0	1	1	1	1	1	1	1	1	1	1	1	1	1	1	1	1	1	1	1
		chuS			Z4910	A	1	0	0	1	1	1	1	1	1	1	1	1	1	1	1	1	1	1	1	1	1	1
		chuT			Z4913	A	1	0	0	1	1	1	1	1	1	1	1	1	1	1	1	1	1	1	1	1	1	1
		chuU			Z4918	A	1	0	0	1	1	1	1	1	1	1	1	1	1	1	1	1	1	1	1	1	1	1
		chuW			Z4914	A	1	0	0	1	1	1	1	1	1	1	1	1	1	1	1	1	1	1	1	1	1	1
		chuX			Z4915	A	1	0	0	1	1	1	1	1	1	1	1	1	1	1	1	1	1	1	1	1	1	1
		chuY			Z4917	A	1	0	0	1	1	1	1	1	1	1	1	1	1	1	1	1	1	1	1	1	1	1
	Iron-regulated element	*ireA*	*E. coli* CFT073/UPEC	NC_004431/Chromosome	c5174	NA	0	0	0	0	0	0	0	0	0	0	0	0	0	0	0	0	0	0	0	0	0	0
	Iron/manganese transport	*sitA*	*E. coli* O44:H18 str. 042/EAEC	NC_017626/Chromosome	EC042_1521	NA	0	0	0	0	0	0	0	0	0	0	0	0	0	0	0	0	0	0	0	0	0	0
		sitB			EC042_1520	NA	0	0	0	0	0	0	0	0	0	0	0	0	0	0	0	0	0	0	0	0	0	0
		sitC			EC042_1519	NA	0	0	0	0	0	0	0	0	0	0	0	0	0	0	0	0	0	0	0	0	0	0
		sitD			EC042_1518	NA	0	0	0	0	0	0	0	0	0	0	0	0	0	0	0	0	0	0	0	0	0	0
	Salmochelin siderophore	*iroB*	*E. coli* str. UM146/AIEC	NC_017632/Chromosome	UM146_12295	A	1	0	0	1	1	1	1	1	1	0	1	1	1	1	1	1	1	1	1	1	1	1
		iroC			UM146_12300	NA	0	0	0	0	0	0	0	0	0	0	0	0	0	0	0	0	0	0	0	0	0	0
		iroD			UM146_12305	NA	0	0	0	0	0	0	0	0	0	0	0	0	0	0	0	0	0	0	0	0	0	0
		iroE			UM146_12310	NA	0	0	0	0	0	0	0	0	0	0	0	0	0	0	0	0	0	0	0	0	0	0
		iroN			UM146_12315	NA	0	0	0	0	0	0	0	0	0	0	0	0	0	0	0	0	0	1	1	1	0	0
	Yersiniabactin siderophore	*fyuA*	*E. coli* O45:K1:H7 str. S88/NMEC	NC_011742/Chromosome	ECS88_2043	NA	0	0	0	0	0	0	0	0	0	0	0	0	0	0	0	0	0	0	0	0	0	0
		irp1			ECS88_2038	NA	0	0	0	0	0	0	0	0	0	0	0	0	0	0	0	0	0	0	0	0	0	0
		irp2			ECS88_2037	NA	0	0	0	0	0	0	0	0	0	0	0	0	0	0	0	0	0	0	0	0	0	0
		ybtA			ECS88_2036	NA	0	0	0	0	0	0	0	0	0	0	0	0	0	0	0	0	0	0	0	0	0	0
		ybtE			ECS88_2041	NA	0	0	0	0	0	0	0	0	0	0	0	0	0	0	0	0	0	0	0	0	0	0
		ybtP			ECS88_2035	NA	0	0	0	0	0	0	0	0	0	0	0	0	0	0	0	0	0	0	0	0	0	0
		ybtQ			ECS88_2034	NA	0	0	0	0	0	0	0	0	0	0	0	0	0	0	0	0	0	0	0	0	0	0
		ybtS			ECS88_2032	NA	0	0	0	0	0	0	0	0	0	0	0	0	0	0	0	0	0	0	0	0	0	0
		ybtT			ECS88_2040	NA	0	0	0	0	0	0	0	0	0	0	0	0	0	0	0	0	0	0	0	0	0	0
		ybtU			ECS88_2039	NA	0	0	0	0	0	0	0	0	0	0	0	0	0	0	0	0	0	0	0	0	0	0
		ybtX			ECS88_2033	NA	0	0	0	0	0	0	0	0	0	0	0	0	0	0	0	0	0	0	0	0	0	0

LEE-encoded T3SS effectors	EspB	*espB*	*E. coli* O157:H7 str. EDL933/EHEC	NC_002655/Chromosome	Z5105	C	1	1	1	1	1	1	1	1	1	1	1	1	1	1	1	1	1	1	1	1	1	1
	EspF	*espF*			Z5100	A	1	1	0	0	1	1	1	1	0	0	1	1	1	1	1	1	1	1	1	1	1	1
	EspG	*espG*			Z5142	A	1	1	0	1	1	1	1	1	1	1	1	1	1	1	1	1	1	1	1	1	1	1
	EspH	*espH*			Z5115	A	1	1	0	1	1	1	1	1	1	1	1	1	1	1	1	1	1	1	1	1	1	1
	Mitochondria-associated protein Map	*map*			Z5113	C	1	1	1	1	1	1	1	1	1	1	1	1	1	1	1	1	1	1	1	1	1	1
	SepZ/EspZ	*EspZ*			Z5122	C	1	1	1	1	1	1	1	1	1	1	1	1	1	1	1	1	1	1	1	1	1	1
	Tir	*tir*			Z5112	A	1	1	0	1	1	1	1	1	0	1	1	1	0	1	1	1	1	1	1	0	1	1
Non-LEE encoded T3SS effectors	Cell-cycle-inhibitory factor Cif	*cif*	*E. coli* O127:H6 str. E2348/69/EPEC	NC_011601/Chromosome	E2348C_0719	A	0	0	0	1	1	1	1	1	1	1	1	1	1	1	1	1	1	1	1	1	1	1
	EspFu/TccP (Tir cytoskeleton coupling protein)	*espFu/tccP*	*E. coli* O157:H7 str. EDL933/EHEC	NC_002655/Chromosome	Z3072	A	1	0	0	0	0	0	0	0	0	0	0	0	0	0	0	0	0	0	0	0	0	0
	EspG2 (EPEC EspC island)	*espG2*	*E. coli* O127:H6 str. E2348/69/EPEC	NC_011601/Chromosome	E2348C_2916	NA	0	0	0	0	0	0	0	0	0	0	0	0	0	0	0	0	0	0	0	0	0	0
	EspJ	*espJ*	*E. coli* O157:H7 str. EDL933/EHEC	NC_002655/Chromosome	Z3071	C	1	2	2	1	1	1	1	1	1	1	1	1	1	1	1	1	1	1	1	1	1	1
	EspK	*espK*			Z1829	C	1	1	1	1	1	1	1	1	1	1	1	1	1	1	1	1	1	1	1	1	1	1
	EspL1	*espL1*			Z2749	C	1	1	1	1	1	1	1	1	1	1	1	1	1	1	1	1	1	1	1	1	1	1
	EspL2	*espL2*			Z4326	A	1	2	2	1	1	1	1	1	1	1	1	1	1	1	1	1	1	1	1	0	1	1
	EspL4	*espL4*			Z5608	C	1	1	1	1	1	1	1	1	1	1	1	1	1	1	1	1	1	1	1	1	1	1
	EspM1	*espM1*			Z2565	C	1	1	1	1	1	1	1	1	1	1	1	1	1	1	1	1	1	1	1	1	1	1
	EspM2	*espM2*			Z3918	A	1	1	1	0	0	0	0	0	0	0	0	0	0	0	0	0	0	0	0	0	0	0
	EspN	*espN*			Z1824	A	0	0	0	1	1	1	1	1	1	0	1	1	0	0	1	1	1	0	1	0	0	0
	EspO1-1	*espO1-1*	*E. coli* O157:H7 str. Sakai/EHEC	NC_002695	ECs1567	A	1	0	0	1	1	1	1	1	1	1	1	1	1	1	1	1	1	1	1	1	1	1
	EspO1-2	*espO1-2*			ECs1821	C	1	1	1	1	1	1	1	1	1	1	1	1	1	1	1	1	1	1	1	1	1	1
	EspR1	*espR1*	*E. coli* O157:H7 str. EDL933/EHEC	NC_002655/Chromosome	Z2242	A	1	0	0	1	1	1	1	1	1	1	1	1	1	1	1	1	1	1	1	1	1	1
	EspR3	*espR3*			Z3023	A	1	0	0	0	0	0	0	0	0	0	0	0	0	0	0	0	0	0	0	0	0	0
	EspR4	*espR4*			Z3026	A	1	0	0	0	0	0	0	0	0	0	0	0	0	0	0	0	0	0	0	0	0	0
	EspV	*espV*			Z1387	C	1	1	1	2	2	1	2	1	2	1	2	2	2	2	1	1	1	1	2	2	1	2
	EspW	*espW*			Z3920	A	1	0	1	0	0	0	0	0	0	0	0	0	0	0	0	0	0	0	0	0	0	0
	EspX1	*espX1*			Z0025	A	1	0	0	1	1	1	1	1	1	1	1	1	1	1	1	1	1	1	1	1	1	1
	EspX2	*espX2*			Z1019	A	1	0	0	1	1	1	1	1	1	1	1	1	1	1	1	1	1	1	1	1	1	1
	EspX4	*espX4*			Z5636	A	1	0	0	1	1	1	1	1	1	1	0	0	0	1	1	1	1	1	1	0	1	1
	EspX5	*espX5*			Z5665	C	1	1	1	1	1	1	1	1	1	1	1	1	1	1	1	1	1	1	1	1	1	1
	EspX6	*espX6*			Z5935	A	1	0	0	1	0	1	1	1	1	1	1	1	1	1	1	1	1	1	1	1	1	1
	EspX7	*espX7*			Z1822	A	1	1	1	1	1	1	1	1	1	1	1	1	1	1	1	1	0	0	1	0	0	0
	EspY1	*espY1*			Z0065	A	1	0	0	1	1	1	1	1	1	1	1	1	1	1	1	1	1	1	1	1	1	1
	EspY2	*espY2*			Z0078	A	1	0	0	1	1	1	1	1	1	1	1	1	1	1	1	1	1	1	1	1	1	1
	EspY3	*espY3*			Z0521	A	1	0	0	1	1	1	1	1	1	1	1	1	1	1	1	1	1	1	1	1	1	1
	EspY4	*espY4*			Z5211	A	1	0	0	1	1	1	1	1	1	1	1	1	1	1	1	1	1	1	1	0	1	1
	EspY5	*espY5*			Z5214	A	1	0	0	0	1	0	0	0	0	0	0	0	0	0	0	0	0	0	0	0	0	0
	LifA/Efa1	*lifA/efa1*			Z4332	A	1	2	2	1	1	0	1	1	1	0	1	1	1	1	0	0	0	0	1	0	0	1
	LifA/Efa2	*lifA/efa1*			Z4333	A	1	2	2	1	1	0	1	1	1	0	1	1	1	1	0	0	0	0	1	0	0	1
	NleA	*nleA*			Z6024	C	1	1	1	1	1	1	1	1	1	1	1	1	1	1	1	1	1	1	1	1	1	1
	NleB1	*nleB1*			Z4328	A	1	2	2	1	1	1	1	1	1	1	1	1	1	1	1	1	1	1	1	0	1	1
	NleB2-1	*nleB2-1*	*E. coli* O103:H2 str. 12009/EHEC	NC_013353/Chromosome	ECO103_2227	A	1	0	0	1	1	1	1	0	1	1	1	1	1	0	1	1	1	1	1	1	1	1
	NleB2-2	*nleB2-2*			ECO103_0801	NA	0	0	0	0	0	0	0	0	0	0	0	0	0	0	0	0	0	0	0	0	0	0
	NleC	*nleC*	*E. coli* O157:H7 str. EDL933/EHEC	NC_002655/Chromosome	Z0986	A	1	3	3	0	1	3	4	1	1	2	2	2	2	1	2	2	2	2	2	2	3	2
	NleD	*nleD*			Z0990	A	1	0	0	0	0	0	0	0	0	0	0	0	0	0	0	0	0	0	0	0	0	0
	NleE-1	*nleE-1*			Z4329	A	1	2	2	1	1	1	1	1	1	1	1	1	1	1	1	1	1	1	1	0	1	1
	NleE-2	*nleE-2*	*E. coli* O127:H6 str. E2348/69/EPEC	NC_011601/Chromosome	E2348C_1080	NA	0	0	0	0	0	0	0	0	0	0	0	0	0	0	0	0	0	0	0	0	0	0
	NleF	*nleF*	*E. coli* O157:H7 str. EDL933/EHEC	NC_002655/Chromosome	Z6020	A	1	1	1	1	1	1	1	0	1	1	1	1	1	0	1	1	1	1	1	1	1	1
	NleG-1	*nleG-1*			Z6010	C	1	2	2	1	1	1	1	1	1	1	1	1	1	1	1	1	1	1	1	1	1	1
	NleG-2	*nleG-2*	*E. coli* O55:H7 str. CB9615/EPEC	NC_013941/Chromosome	G2583_0341	A	0	2	2	0	0	0	0	0	0	0	0	0	0	0	0	0	0	0	0	0	0	0
	NleG-3	*nleG-3*	*E. coli* O157:H7 str. EC4115/EHEC	NC_011353/Chromosome	ECH74115_5546	A	0	0	0	0	0	0	0	0	0	0	0	0	0	0	0	0	0	0	0	0	0	0
	NleG2-2	*nleG2-2*	*E. coli* O157:H7 str. EDL933/EHEC	NC_002655/Chromosome	Z2339	A	1	0	0	0	0	0	0	0	0	0	0	0	0	0	0	0	0	0	0	0	0	0
	NleG2-3	*nleG2-3*			Z2149	C	1	1	1	0	0	0	0	0	0	0	0	0	0	0	0	0	0	0	0	0	0	0
	NleG2-4	*nleG2-4*			Z2075	A	1	1	1	0	0	0	0	0	0	0	0	0	0	0	0	0	0	0	0	0	0	0
	NleG5-1	*nleG5-1*			Z2337	A	1	1	1	0	0	0	0	0	0	0	0	0	0	0	0	0	0	0	0	0	0	0
	NleG5-2	*nleG5-2*			Z2151	A	1	0	0	0	0	0	0	0	0	0	0	0	0	0	0	0	0	0	0	0	0	0
	NleG6-1	*nleG6-1*			Z2338	A	1	1	1	0	0	0	0	0	0	0	0	0	0	0	0	0	0	0	0	0	0	0
	NleG6-2	*nleG6-2*			Z2150	A	1	1	1	0	0	0	0	0	0	0	0	0	0	0	0	0	0	0	0	0	0	0
	NleG6-3	*nleG6-3*			Z3921	A	1	0	0	0	0	0	0	0	0	0	0	0	0	0	0	0	0	0	0	0	0	0
	NleG7	*nleG7*			Z2077	C	1	1	1	2	2	1	1	1	1	1	1	1	1	2	1	1	1	1	1	1	1	1
	NleG8-2	*nleG8-2*			Z3919	C	1	2	2	1	1	1	1	1	1	1	1	1	1	1	1	1	1	1	1	1	1	1
	NleH1-1	*nleH1-1*			Z0989	C	1	1	1	1	1	1	1	1	1	1	1	1	1	1	1	1	1	1	1	1	1	1
	NleH1-2	*nleH1-2*			Z6021	A	1	1	1	1	1	1	1	0	1	1	1	1	1	0	1	1	1	1	1	1	1	1
	TccP2	*tccP2*			Z1385	A	1	0	0	1	0	0	1	1	1	1	1	1	1	1	1	1	1	1	1	1	1	1
Secretion system	T6SS-3	*aaiA*	*E. coli* O104:H4 str. 2011C-3493 /StxEAEC	NC_018658/Chromosome	O3K_04355	NA	0	0	0	0	0	0	0	0	0	0	0	0	0	0	0	0	0	0	0	0	0	0
		aaiB			O3K_04360	NA	0	0	0	0	0	0	0	0	0	0	0	0	0	0	0	0	0	0	0	0	0	0
		aaiC/hcp			O3K_04365	NA	0	0	0	0	0	0	0	0	0	0	0	0	0	0	0	0	0	0	0	0	0	0
		aaiD			O3K_04370	NA	0	0	0	0	0	0	0	0	0	0	0	0	0	0	0	0	0	0	0	0	0	0
		aaiE			O3K_04380	NA	0	0	0	0	0	0	0	0	0	0	0	0	0	0	0	0	0	0	0	0	0	0
		aaiF			O3K_04385	NA	0	0	0	0	0	0	0	0	0	0	0	0	0	0	0	0	0	0	0	0	0	0
		aaiH			O3K_04395	NA	0	0	0	0	0	0	0	0	0	0	0	0	0	0	0	0	0	0	0	0	0	0
		aaiI			O3K_04400	NA	0	0	0	0	0	0	0	0	0	0	0	0	0	0	0	0	0	0	0	0	0	0
		aaiJ			O3K_04405	NA	0	0	0	0	0	0	0	0	0	0	0	0	0	0	0	0	0	0	0	0	0	0
		aaiK			O3K_04410	NA	0	0	0	0	0	0	0	0	0	0	0	0	0	0	0	0	0	0	0	0	0	0
		aaiL			O3K_04415	NA	0	0	0	0	0	0	0	0	0	0	0	0	0	0	0	0	0	0	0	0	0	0
		aaiM			O3K_04420	NA	0	0	0	0	0	0	0	0	0	0	0	0	0	0	0	0	0	0	0	0	0	0
		aaiN			O3K_04425	NA	0	0	0	0	0	0	0	0	0	0	0	0	0	0	0	0	0	0	0	0	0	0
		clpV/aaiP			O3K_04435	NA	0	0	0	0	0	0	0	0	0	0	0	0	0	0	0	0	0	0	0	0	0	0

		icmF/aaiO			O3K_04430	NA	0	0	0	0	0	0	0	0	0	0	0	0	0	0	0	0	0	0	0	0	0	0
		vgrG			O3K_04390	NA	0	0	0	0	0	0	0	0	0	0	0	0	0	0	0	0	0	0	0	0	0	0
	ABC transporter for dispersin	*aatA*	*E. coli* O104:H4 str. 2011C-3493 /StxEAEC	NC_018666/Chromosome	O3K_26477	NA	0	0	0	0	0	0	0	0	0	0	0	0	0	0	0	0	0	0	0	0	0	0
		aatB			O3K_26472	NA	0	0	0	0	0	0	0	0	0	0	0	0	0	0	0	0	0	0	0	0	0	0
		aatC			O3K_26467	NA	0	0	0	0	0	0	0	0	0	0	0	0	0	0	0	0	0	0	0	0	0	0
		aatD			O3K_26462	NA	0	0	0	0	0	0	0	0	0	0	0	0	0	0	0	0	0	0	0	0	0	0
		aatP			O3K_26482	NA	0	0	0	0	0	0	0	0	0	0	0	0	0	0	0	0	0	0	0	0	0	0
	T6SS-2	*tssF*	*E. coli* O45:K1:H7 str. S88/NMEC	NC_011742/Chromosome	ECS88_0243	A	1	1	1	1	1	1	1	1	0	1	1	1	1	1	1	1	1	1	1	1	1	1
		tssI			ECS88_1552	C	1	1	1	1	1	1	1	1	1	1	1	1	1	1	1	1	1	1	1	1	1	1
		aec11			ECS88_0251	A	0	0	0	1	1	1	1	1	1	1	1	1	1	1	1	1	1	1	1	1	1	1
		aec14			ECS88_0249	NA	0	0	0	0	0	0	0	0	0	0	0	0	0	0	0	0	0	0	0	0	0	0
		aec15			ECS88_0248	C	2	1	1	0	0	0	0	0	0	0	0	0	0	0	0	0	0	0	0	0	0	0
		aec16			ECS88_0247	A	1	0	0	0	0	0	0	0	0	0	0	0	0	0	0	0	0	0	0	0	0	0
		aec17			ECS88_0246	NA	0	0	0	0	0	0	0	0	0	0	0	0	0	0	0	0	0	0	0	0	0	0
		aec18			ECS88_0245	NA	0	0	0	0	0	0	0	0	0	0	0	0	0	0	0	0	0	0	0	0	0	0
		aec19			ECS88_0244	NA	0	0	0	0	0	0	0	0	0	0	0	0	0	0	0	0	0	0	0	0	0	0
		aec22			ECS88_0242	A	1	0	0	1	1	1	1	1	1	1	1	1	1	1	1	1	1	1	1	1	1	1
		aec23			ECS88_0241	A	1	0	0	0	0	0	0	0	0	0	0	0	0	0	0	0	0	0	0	0	0	0
		aec24			ECS88_0240	A	1	0	0	0	0	0	0	0	0	0	0	0	0	0	0	0	0	0	0	0	0	0
		aec25			ECS88_0239	A	1	0	0	0	0	0	0	0	0	0	0	0	0	0	0	0	0	0	0	0	0	0
		aec26			ECS88_0238	A	1	0	0	0	0	0	0	0	0	0	0	0	0	0	0	0	0	0	0	0	0	0
		aec27/clpV			ECS88_0237	A	1	1	1	0	0	0	0	0	0	0	0	0	0	0	0	0	0	0	0	0	0	0
		aec28			ECS88_0236	A	1	1	1	0	0	0	0	0	0	0	0	0	0	0	0	0	0	0	0	0	0	0
		aec29			ECS88_0235	A	0	1	1	0	0	0	0	0	0	0	0	0	0	0	0	0	0	0	0	0	0	0
		aec30			ECS88_0234	A	1	1	1	0	0	0	0	0	0	0	0	0	0	0	0	0	0	0	0	0	0	0
		aec31			ECS88_0233	A	1	0	0	0	0	0	0	0	0	0	0	0	0	0	0	0	0	0	0	0	0	0
		aec32			ECS88_0232	A	1	1	1	1	1	1	1	1	1	1	1	1	1	1	1	1	1	1	1	0	1	1
		aec7			ECS88_0255	NA	0	0	0	0	0	0	0	0	0	0	0	0	0	0	0	0	0	0	0	0	0	0
		aec8			ECS88_0254	NA	0	0	0	0	0	0	0	0	0	0	0	0	0	0	0	0	0	0	0	0	0	0
	LEE locus encoded T3SS	NA	*E. coli* O157:H7 str. EDL933/EHEC	NC_002655/Chromosome	Z5102	C	1	1	1	1	1	1	1	1	1	1	1	1	1	1	1	1	1	1	1	1	1	1
		NA			Z5121	C	1	1	1	1	1	1	1	1	1	1	1	1	0	1	1	1	1	1	1	1	1	1
		cesAB			Z5138	C	1	1	1	1	1	1	1	1	1	1	1	1	1	1	1	1	1	1	1	1	1	1
		NA			Z5139	C	1	1	1	1	1	1	1	1	1	1	1	1	1	1	1	1	1	1	1	1	1	1
		NA			Z5143	C	1	1	1	1	1	1	1	1	1	1	1	1	1	1	1	1	1	1	1	1	1	1
		cesD2			Z5104	C	1	1	1	1	1	1	1	1	1	1	1	1	1	1	1	1	1	1	1	1	1	1
		cesD			Z5127	C	1	1	1	1	1	1	1	1	1	1	1	1	1	1	1	1	1	1	1	1	1	1
		cesF			Z5114	C	1	1	1	1	1	1	1	1	1	1	1	1	1	1	1	1	1	1	1	1	1	1
		cesT			Z5111	C	1	1	1	1	1	1	1	1	1	1	1	1	1	1	1	1	1	1	1	1	1	1
		escC			Z5126	C	1	1	1	1	1	1	1	1	1	1	1	1	1	1	1	1	1	1	1	1	1	1
		escD			Z5109	C	1	1	1	1	1	1	1	1	1	1	1	1	1	1	1	1	1	1	1	1	1	1
		escF			Z5103	C	1	1	1	1	1	1	1	1	1	1	1	1	1	1	1	1	1	1	1	1	1	1
		escI			Z5123	C	1	1	1	1	1	1	1	1	1	1	1	1	1	1	1	1	1	1	1	1	1	1
		escJ			Z5124	C	1	1	1	1	1	1	1	1	1	1	1	1	1	1	1	1	1	1	1	1	1	1
		escK			Z5137	C	1	1	1	1	1	1	1	1	1	1	1	1	1	1	1	1	1	1	1	1	1	1
		escL			Z5136	C	1	1	1	1	1	1	1	1	1	1	1	1	1	1	1	1	0	1	1	1	1	1
		escN			Z5119	C	1	1	0	1	1	1	1	1	1	0	0	1	1	1	1	1	1	0	1	0	1	0
		escO			Z5118	C	1	1	1	1	1	1	1	1	1	1	1	1	1	1	1	1	1	1	1	1	1	1
		escP			Z5117	C	1	1	1	1	1	1	1	1	1	1	1	1	1	1	1	1	1	1	1	1	1	1
		escR			Z5135	C	1	1	1	1	1	1	1	1	1	1	1	1	0	1	1	1	1	1	1	1	1	1
		escS			Z5134	C	1	1	1	1	1	1	1	1	1	1	1	1	1	1	1	1	1	1	1	1	1	1
		escT			Z5133	C	1	1	1	1	1	1	1	1	1	1	1	1	1	1	1	1	1	1	1	1	1	1
		escU			Z5132	C	1	1	1	1	1	1	1	1	1	1	1	1	1	1	1	1	0	1	1	1	1	1
		escV			Z5120	C	1	1	1	1	1	1	1	1	1	1	1	1	1	1	1	1	1	1	1	1	1	1
		espA			Z5107	C	1	1	1	1	1	1	1	1	1	1	1	1	1	1	1	1	1	1	1	1	1	1
		espB			Z5105	C	1	1	1	1	1	1	1	1	1	1	1	1	1	1	1	1	1	1	1	1	1	1
		espD			Z5106	C	1	1	1	1	1	1	1	1	1	1	1	1	1	1	1	1	1	1	1	1	1	1
		etgA			Z5131	C	1	1	1	1	1	1	1	1	1	1	1	1	1	1	1	1	1	1	1	1	1	1
		glrA			Z5128	C	1	1	1	1	1	1	1	1	1	1	1	1	1	1	1	1	1	1	1	1	1	1
		glrR			Z5129	C	1	1	1	1	1	1	1	1	1	1	1	1	1	1	1	1	1	1	1	0	1	1
		ler			Z5140	C	1	1	1	1	1	1	1	1	1	1	1	1	1	1	1	1	1	1	1	1	1	1
		sepD			Z5125	C	1	1	1	1	1	1	1	1	1	1	1	1	1	1	1	1	1	1	1	1	1	1
		sepL			Z5108	C	1	1	1	1	1	1	1	1	1	1	1	1	1	1	1	1	1	1	1	1	1	1
		sepQ			Z5116	C	1	1	1	1	1	1	1	1	1	1	1	1	1	1	1	1	1	1	1	1	1	1
	T6SS-1	NA	*E. coli* O44:H18 str. 042/EAEC	NC_017626/Chromosome	EC042_4524	NA	0	0	0	0	0	0	0	0	0	0	0	0	0	0	0	0	0	0	0	0	0	0
		NA			EC042_4525	NA	0	0	0	0	0	0	0	0	0	0	0	0	0	0	0	0	0	0	0	0	0	0
		NA			EC042_4526	NA	0	0	0	0	0	0	0	0	0	0	0	0	0	0	0	0	0	0	0	0	0	0
		NA			EC042_4527	NA	0	0	0	0	0	0	0	0	0	0	0	0	0	0	0	0	0	0	0	0	0	0
		NA			EC042_4528	NA	0	0	0	0	0	0	0	0	0	0	0	0	0	0	0	0	0	0	0	0	0	0
		NA			EC042_4529	NA	0	0	0	0	0	0	0	0	0	0	0	0	0	0	0	0	0	0	0	0	0	0
		NA			EC042_4530	NA	0	0	0	0	0	0	0	0	0	0	0	0	0	0	0	0	0	0	0	0	0	0
		NA			EC042_4531	NA	0	0	0	0	0	0	0	0	0	0	0	0	0	0	0	0	0	0	0	0	0	0
		NA			EC042_4532	NA	0	0	0	0	0	0	0	0	0	0	0	0	0	0	0	0	0	0	0	0	0	0
		NA			EC042_4533	NA	0	0	0	0	0	0	0	0	0	0	0	0	0	0	0	0	0	0	0	0	0	0
		NA			EC042_4534	NA	0	0	0	0	0	0	0	0	0	0	0	0	0	0	0	0	0	0	0	0	0	0
		NA			EC042_4535	NA	0	0	0	0	0	0	0	0	0	0	0	0	0	0	0	0	0	0	0	0	0	0
		NA			EC042_4536	NA	0	0	0	0	0	0	0	0	0	0	0	0	0	0	0	0	0	0	0	0	0	0
		NA			EC042_4537	NA	0	0	0	0	0	0	0	0	0	0	0	0	0	0	0	0	0	0	0	0	0	0
		NA			EC042_4538	NA	0	0	0	0	0	0	0	0	0	0	0	0	0	0	0	0	0	0	0	0	0	0
		NA			EC042_4539	NA	0	0	0	0	0	0	0	0	0	0	0	0	0	0	0	0	0	0	0	0	0	0
		NA			EC042_4540	NA	0	0	0	0	0	0	0	0	0	0	0	0	0	0	0	0	0	0	0	0	0	0
		NA			EC042_4541	NA	0	0	0	0	0	0	0	0	0	0	0	0	0	0	0	0	0	0	0	0	0	0

		NA			EC042_4542	NA	0	0	0	0	0	0	0	0	0	0	0	0	0	0	0	0	0	0	0	0	0	0
		NA			EC042_4543	NA	0	0	0	0	0	0	0	0	0	0	0	0	0	0	0	0	0	0	0	0	0	0
		NA			EC042_4544	NA	0	0	0	0	0	0	0	0	0	0	0	0	0	0	0	0	0	0	0	0	0	0
		NA			EC042_4545	NA	0	0	0	0	0	0	0	0	0	0	0	0	0	0	0	0	0	0	0	0	0	0
		NA			EC042_4546	NA	0	0	0	0	0	0	0	0	0	0	0	0	0	0	0	0	0	0	0	0	0	0
		NA			EC042_4547	NA	0	0	0	0	0	0	0	0	0	0	0	0	0	0	0	0	0	0	0	0	0	0
		NA			EC042_4548	NA	0	0	0	0	0	0	0	0	0	0	0	0	0	0	0	0	0	0	0	0	0	0
		NA			EC042_4549	NA	0	0	0	0	0	0	0	0	0	0	0	0	0	0	0	0	0	0	0	0	0	0
		NA			EC042_4550	NA	0	0	0	0	0	0	0	0	0	0	0	0	0	0	0	0	0	0	0	0	0	0
Toxin	Alpha-hemolysin	*hlyA*	*E. coli* O157:H7 str. EDL933/EHEC	NC_007414/Plasmid pO157	L7048	A	1	1	1	1	1	1	1	1	0	1	1	1	1	1	1	1	1	1	1	1	1	1
		hlyB			L7049	A	1	1	1	1	1	1	1	1	0	0	1	1	1	1	1	1	1	1	1	1	1	1
		hlyC			L7047	A	1	1	1	1	1	1	1	1	0	0	1	1	1	1	1	0	1	1	1	1	1	1
		hlyD			L7050	A	1	1	1	1	1	1	1	1	0	1	1	1	1	1	1	1	1	1	1	1	1	1
	Colicin-like Usp	*hcp*	*E. coli* str. UM146/AIEC	NC_017632/Chromosome	UM146_23345	NA	0	0	0	0	0	0	0	0	0	0	0	0	0	0	0	0	0	0	0	0	0	0
	Cytolethal distending toxin	*cdtA*	*E. coli* O18:K1:H7 str. IHE3034/NMEC	NC_017628/Chromosome	ECOK1_2089	NA	0	0	0	0	0	0	0	0	0	0	0	0	0	0	0	0	0	0	0	0	0	0
		cdtB			ECOK1_2088	NA	0	0	0	0	0	0	0	0	0	0	0	0	0	0	0	0	0	0	0	0	0	0
		cdtC			ECOK1_2087	NA	0	0	0	0	0	0	0	0	0	0	0	0	0	0	0	0	0	0	0	0	0	0
	Cytotoxic necrotizing factor 1	*cnf1*	*E. coli* str. UM146/AIEC	NC_017632/Chromosome	UM146_21825	NA	0	0	0	0	0	0	0	0	0	0	0	0	0	0	0	0	0	0	0	0	0	0
	Enterotoxin 1	*pic*	*E. coli* O44:H18 str. 042/EAEC	NC_017626/Chromosome	EC042_4593	NA	0	0	0	0	0	0	0	0	0	0	0	0	0	0	0	0	0	0	0	0	0	0
	Enterotoxin SenB/TieB	*senB*	*E. coli* str. UM146/AIEC	NC_017630/Chromosome	UM146_24581	NA	0	0	0	0	0	0	0	0	0	0	0	0	0	0	0	0	0	0	0	0	0	0
	Heat-labile enterotoxin	*eltA*	*E. coli* O78:H11:K80 str. H10407/ETEC	NC_017722/Plasmid p666	ETEC_p666_0660	NA	0	0	0	0	0	0	0	0	0	0	0	0	0	0	0	0	0	0	0	0	0	0
		eltB			ETEC_p666_0650	NA	0	0	0	0	0	0	0	0	0	0	0	0	0	0	0	0	0	0	0	0	0	0
	Hemolysin/cytolysin A	*hlyE/clyA*	*E. coli* O157:H7 str. EDL933/EHEC	NC_002655/Chromosome	Z1944	A	1	0	0	1	1	1	1	1	1	1	1	1	1	1	1	1	1	1	1	1	1	1
	Shiga-like toxin	*stx1A*	*E. coli* O157:H7 str. EDL933/EHEC	NC_002655/Chromosome	Z3344	A	1	0	0	0	0	1	1	0	0	1	1	1	1	0	0	0	0	0	1	1	0	1
		stx1B			Z3343	A	1	0	0	0	0	1	1	0	0	1	1	1	1	0	0	0	0	0	1	1	0	1
		stx2A	*E. coli* O157:H7 str. EDL933/EHEC	NC_002655/Chromosome	Z1464	A	1	1	1	1	1	1	2	1	1	1	0	0	0	1	1	1	1	1	0	0	2	0
		stx2B			Z1465	A	1	2	1	1	1	1	2	1	1	1	0	0	0	1	1	1	1	1	0	0	2	0

[1]The number ≥1 refers to the wild-type (WT) gene and the number 0 indicates that either the gene is absent or carries a loss-of-function mutation. [2]NA, gene name is not available in the published genome annotations; [3]NA, not applicable; A, accessory gene; C, core gene; [4]Genome of strain 95-3192 in GenBank does not contain a pEHEC plasmid;
