# Supplementary material for: Comparative Genomics Applied to Systematically Assess Pathogenicity Potential in Shiga Toxin-Producing Escherichia coli O145:H28
Source: Microorganisms. 2022 Apr 21;10(5):866. doi: 10.3390/microorganisms10050866 (PMC9144400; doi:10.3390/microorganisms10050866)
Supplement: Supplementary file 1 [file microorganisms-10-00866-s001.zip › Table S2.pdf]

**Table S2. Mutations in virulence genes**

| VFclass | Virulence factors | Genes | EDL933 | CFSAN004176 | CFSAN004177 | RM13514 | RM13516 | 10942 | 112648 | 122715 | 95-3192* | 2015C-3125 | RM8843-C1 |
|---|---|---|---|---|---|---|---|---|---|---|---|---|---|
| Adherence | Curli | *csgB* | WT | WT | WT | WT | WT | WT | Nonsense mutation | WT | WT | WT | WT |
| | ECP | *ecpE* | WT | WT | Point deletion | WT | WT | WT | WT | WT | WT | WT | WT |
| | ELF | *elfC* | Point deletion | WT | Point deletion | WT | WT | WT | WT | WT | WT | Point deletion | Point deletion |
| | | *elfD* | WT | WT | Point deletion | WT | WT | WT | WT | WT | WT | WT | WT |
| | | *elfG* | WT | WT | Point deletion | WT | WT | WT | WT | WT | Point deletion | WT | WT |
| | EaeH/Intimin like adhesin FdeC | *eaeH* | WT | WT | WT | WT | A | WT | WT | WT | WT | WT | WT |
| | Intimin | *eae* | WT | WT | WT | WT | WT | WT | WT | WT | Point deletion | WT | WT |
| | ToxB | *toxB* | WT | A | A | WT | A | WT | WT | WT | NA | Point deletion | WT |
| | Type I fimbriae | *fimB* | WT | WT | WT | WT | Point deletion | WT | WT | WT | WT | WT | WT |
| | | *fimD* | WT | WT | WT | WT | WT | WT | WT | WT | WT | Point deletion | WT |
| Autotransporter | Antigen 43 | *ag43* | A | A | A | WT | 1+0 (Point insertion) | A | WT | WT | WT | Point deletion | WT |
| | Cah | *cah* | WT | A | A | Transposon insertion | Large deletion | WT | WT | WT | WT | WT | WT |
| | EhaA | *ehaA* | WT | WT | WT | WT | WT | WT | WT | WT | WT | WT | WT |
| | EhaB | *ehaB* | WT | WT | WT | WT | WT | Transposon insertion | WT | WT | WT | WT | WT |
| | EspP | *espP* | WT | A | A | WT | A | WT | WT | WT | A | A | WT |
| | UpaG adhesin | *upaG/ehaG* | WT | WT | WT | WT | WT | Point insertion | WT | WT | WT | WT | WT |
| Invasion | IbeC | *ibeC* | WT | WT | WT | WT | WT | WT | WT | WT | WT | 1+0 (Point deletion) | WT |
| Iron uptake | Aerobactin siderphore | *iucC* | A | A | A | WT | WT | WT | WT | WT | WT | WT | WT |
| | | *iucD* | A | A | A | WT | WT | WT | WT | WT | WT | WT | WT |
| | Salmochelin siderophore | *iroB* | 1+0 (Nonsense mutation) | A | A | WT | WT | WT | WT | WT | WT | Point deletion | WT |
| LEE encoded TTSS effectors | EspF | *espF* | WT | WT | Point deletion | Large deletion | WT | WT | WT | WT | Point deletion | Point deletion | WT |
| | EspG | *espG* | WT | WT | Point deletion | WT | WT | WT | WT | WT | WT | WT | WT |
| | EspH | *espH* | WT | WT | Point deletion | WT | WT | WT | WT | WT | WT | WT | WT |
| | Tir | *tir* | WT | WT | Point deletion | WT | WT | WT | WT | WT | Point deletion | WT | WT |
| nonLEE encoded TTSS effectors | EspN | *espN* | Point insertion | Point deletion | Point deletion | WT | WT | WT | WT | WT | WT | Point deletion | WT |
| | EspV | *espV* | WT | WT | WT | WT | WT | 1+0 (Transposon insertion) | 1+0 (Nonsense mutaiton) | 1+0 (Transposon insertion) | 1+0 (Transposon insertion ) | 1+0 (Transposon insertion) | 1+0 (Nonsense mutaiton) |
| | EspW | *espW* | WT | Point deletion | WT | A | A | A | A | A | A | A | A |
| | EspX1 | *espX1* | WT | Point deletion | Point deletion | WT | WT | WT | WT | WT | WT | WT | WT |
| | EspX4 | *espX4* | WT | Nonsense mutation | Nonsense mutation | WT | WT | WT | WT | WT | WT | WT | Point deletion |
| | EspX7 | *espX7* | WT | WT | WT | WT | WT | WT | WT | WT | WT | WT | WT |
| | EspY4 | *espY4* | WT | A | A | WT | WT | WT | WT | WT | WT | WT | WT |
| | EspY5 | *espY5* | WT | A | A | Nonsense mutation | WT | Nonsense mutation | Nonsense mutation | Nonsense mutation | Nonsense mutation | Nonsense mutation | Nonsense mutation |
| | NleC | *nleC* | WT | WT | WT | Large insertion | WT | WT | WT | WT | WT | WT | WT |
| | NleG2-3 | *nleG2-3* | WT | WT | WT | Point deletions (2) and insertion (1) | Point deletions (2) and insertion (1) | Point deletions (2) and insertion (1) | Point deletions (2) and insertion (1) | Point deletions (2) and insertion (1) | Point deletions (2) and insertion (1) | Point deletions (2) and insertion (1) | Point deletions (2) and insertion (1) |
| Secretion systems | ACE T6SS | *tssF* | WT | WT | WT | WT | WT | WT | WT | WT | Point deletion | WT | WT |
| | | *aec32* | WT | WT | WT | WT | WT | WT | WT | WT | WT | WT | WT |
| | TTSS | *cesL* | WT | WT | WT | WT | WT | WT | WT | WT | WT | WT | WT |
| | | *escL* | WT | WT | WT | WT | WT | WT | WT | WT | WT | WT | WT |
| | | *escN* | WT | WT | Point deletion | WT | WT | WT | WT | WT | WT | Point deletion | Point deletion |
| | | *escR* | WT | WT | WT | WT | WT | WT | WT | WT | WT | WT | WT |
| | | *escU* | WT | WT | WT | WT | WT | WT | WT | WT | WT | WT | WT |
| | | *glrR* | WT | WT | WT | WT | WT | WT | WT | WT | WT | WT | WT |
| Toxin | Enterohemolysin | *hlyB* | WT | WT | WT | WT | WT | WT | WT | WT | A | Point deletion | WT |
| | | *hlyC* | WT | WT | WT | WT | WT | WT | WT | WT | A | Point deletion | WT |

WT: wild-type; A: absence; NA: not available

When both WT and mutated loci are present, 1 represents WT, while 0 represents loss-of-function mutations.

Point insertion or deletion: insertion or deletion of a single base within the coding sequence;  Large insertion or deletion: insertion or deletion of a oligonucleotide within the coding region.

| RM8988-C1 | RM8995-C1 | RM11626-C1 | RM12275-C1 | RM12522-C8 | RM9154-C1 | RM9467-C1 | RM9872-C1 | RM9873-C1 | RM10425-C1 | RM12367-C1 |
|---|---|---|---|---|---|---|---|---|---|---|
| WT | WT | WT | WT | WT | WT | WT | WT | WT | WT | WT |
| WT | WT | WT | WT | WT | WT | Point deletion | WT | WT | WT | WT |
| WT | Point deletion | WT | Point deletion | WT | WT | WT | WT | WT | WT | Point deletion |
| WT | WT | WT | WT | WT | WT | WT | WT | WT | WT | WT |
| WT | WT | WT | WT | WT | WT | WT | WT | WT | WT | Point deletion |
| WT | Point deletion | WT | Point deletion | WT | WT | WT | WT | Point deletion | WT | WT |
| WT | WT | WT | Point deletion | WT | WT | WT | WT | WT | WT | WT |
| WT | Point deletion | Point deletion | Point deletion | Point deletion | WT | Point deletion | Point deletion | Point deletion | WT | Point deletion |
| WT | WT | WT | WT | WT | WT | WT | WT | WT | WT | WT |
| WT | WT | WT | WT | WT | WT | WT | WT | WT | WT | WT |
| WT | WT | WT | A | WT | A | WT | WT | WT | WT | WT |
| WT | WT | WT | A | WT | WT | Point deletion | WT | Point deletion | WT | WT |
| WT | WT | WT | Point deletion | WT | IS3 insertion | WT | WT | WT | WT | WT |
| WT | WT | WT | WT | WT | WT | WT | WT | WT | WT | WT |
| WT | WT | WT | Point deletion | WT | WT | WT | WT | WT | WT | WT |
| WT | WT | WT | WT | WT | WT | WT | WT | WT | WT | WT |
| WT | WT | WT | WT | WT | WT | WT | WT | WT | WT | WT |
| WT | WT | WT | WT | WT | A | IS3 insertion | IS3 insertion | IS3 insertion | IS3 insertion | IS3 insertion |
| WT | WT | IS629 insertion | WT | IS629 insertion | A | WT | WT | WT | WT | WT |
| WT | WT | WT | WT | WT | WT | WT | WT | WT | WT | WT |
| WT | WT | WT | WT | WT | WT | WT | WT | WT | WT | WT |
| WT | WT | WT | WT | WT | WT | WT | WT | WT | WT | WT |
| WT | WT | WT | WT | WT | WT | WT | WT | WT | WT | WT |
| WT | Point deletion | WT | Point deletion | WT | WT | WT | WT | WT | WT | WT |
| WT | Point deletion | WT | Point deletion | Point deletion | Point deletion | WT | WT | WT | A | Point deletion |
| 1+0 (Nonsense mutaiton) | 1+0 (Nonsense mutation) | 1+0 (Nonsense mutation) | 1+0 (Nonsense mutation) | 1 + 0 (Nonsense mutaiton) | 1+0 (Transposon insertion) | 1+0 (Transposon insertion) | 1+0 (Transposon insertion) | 1+0 (Transposon insertion) | 1+0 (Transposon insertion) | 1+0 (Transposon insertion) |
| A | A | A | A | A | A | A | A | A | A | A |
| WT | WT | WT | WT | WT | WT | WT | WT | WT | WT | WT |
| Point deletion | Point deletion | WT | Point deletion | WT | WT | WT | WT | WT | WT | WT |
| WT | WT | WT | Point deletion | Point deletion | WT | WT | WT | Point deletion | A | Point deletion |
| WT | WT | WT | Point deletion | WT | WT | WT | WT | WT | WT | WT |
| Nonsense mutation | Nonsense mutation | 0 (Nonsense mutation) | 0 (Nonsense mutation) | 0 (Nonsense mutation) | Nonsense mutation | Nonsense mutation | Nonsense mutation | Nonsense mutation | Nonsense mutation | Nonsense mutation |
| WT | WT | WT | WT | WT | WT | WT | WT | WT | WT | WT |
| Point deletions (2) and insertion (1) | Point deletions (2) and insertion (1) | Point deletions (2) and insertion (1) | Point deletions (2) and insertion (1) | Point deletions (2) and insertion (1) | Point deletions (2) and insertion (1) | Point deletions (2) and insertion (1) | Point deletions (2) and insertion (1) | Point deletions (2) and insertion (1) | Point deletions (2) and insertion (1) | Point deletions (2) and insertion (1) |
| WT | WT | WT | WT | WT | WT | WT | WT | WT | WT | WT |
| WT | WT | WT | Point deletion | WT | WT | WT | WT | WT | WT | WT |
| WT | Point deletion | WT | WT | WT | WT | WT | WT | WT | WT | WT |
| WT | WT | WT | WT | WT | WT | WT | WT | Point deletion | WT | WT |
| WT | WT | WT | Point deletion | Point deletion | WT | WT | WT | WT | Point deletion | WT |
| WT | Point deletion | WT | WT | WT | WT | WT | WT | WT | WT | WT |
| WT | WT | WT | WT | WT | WT | WT | WT | Point deletion | WT | WT |
| WT | WT | WT | Point deletion | WT | WT | WT | WT | WT | WT | WT |
| WT | WT | WT | WT | WT | WT | WT | WT | WT | WT | WT |
| WT | WT | WT | WT | WT | WT | WT | Point deletion | WT | WT | WT |
